# Strategies to Treat and Prevent HIV in the United States for Adolescents and Young Adults: Protocol for a Mixed-Methods Study

**DOI:** 10.2196/10759

**Published:** 2019-01-21

**Authors:** Mary Jane Rotheram, Maria Isabel Fernandez, Sung-Jae Lee, Sue Ellen Abdalian, Leslie Kozina, Maryann Koussa, Warren Scott Comulada, Jeffrey D Klausner, Elizabeth Mayfield Arnold, Manuel A Ocasio, Dallas Swendeman, Adolescent Medicine Trials Network (ATN) CARES Team

**Affiliations:** 1 Department of Psychiatry & Biobehavioral Sciences University of California Los Angeles Los Angeles, CA United States; 2 College of Osteopathic Medicine Nova Southeastern University Fort Lauderdale, FL United States; 3 Department of Pediatrics School of Medicine Tulane University New Orleans, LA United States; 4 Division of Infectious Diseases David Geffen School of Medicine University of California Los Angeles Los Angeles, CA United States; 5 UT Southwestern Medical Center Dallas, TX United States; 6 University of California Los Angeles Los Angeles, CA United States; 7 School of Medicine University of California San Francisco San Francisco, CA United States

**Keywords:** gay, bisexual, and transgender youth, HIV/AIDS, homelessness, LGBTQ, mobile phone

## Abstract

**Background:**

Over 20% of HIV diagnoses in the United States are among youth aged 12-24 years. Furthermore, youth have the lowest rates of uptake and adherence to antiretroviral (ARV) medications and are least aware of their HIV status.

**Objective:**

Our objective was to design a set of interrelated studies to promote completion of each step of the HIV Prevention Continuum by uninfected youth at high risk (YHR), as well as completion of steps in the Treatment Continuum by youth living with HIV (YLH).

**Methods:**

Gay, bisexual, and transgender youth; homeless youth; substance-abusing youth; youth with criminal justice contact; and youth with significant mental health challenges, particularly black and Latino individuals, are being recruited from 13 community-based organizations, clinics, drop-in centers, and shelters in Los Angeles and New Orleans. Youth are screened on the basis of self-reports and rapid diagnostic tests for HIV, drug use, and sexually transmitted infections and, then, triaged into one of 3 studies: (1) an observational cohort of YLH who have never received ARV medications and are then treated—half initially are in the acute infection period (n=36) and half with established HIV infection (n=36); (2) a randomized controlled trial (RCT) for YLH (N=220); and (3) an RCT for YHR (N=1340). Each study contrasts efficacy and costs of 3 interventions: an automated messaging and weekly monitoring program delivered via text messages (short message service, SMS); a peer support intervention delivered via social media forums; and coaching, delivered via text message (SMS), phone, and in-person or telehealth contacts. The primary outcomes are assessing youths’ uptake and retention of and adherence to the HIV Prevention or Treatment Continua. Repeat assessments are conducted every 4 months over 24 months to engage and retain youth and to monitor their status.

**Results:**

The project is funded from September 2016 through May 2021. Recruitment began in May 2017 and is expected to be completed by June 2019. We expect to submit the first results for publication by fall 2019.

**Conclusions:**

Using similar, flexible, and adaptable intervention approaches for YLH and YHR, this set of studies may provide a roadmap for communities to broadly address HIV risk among youth. We will evaluate whether the interventions are cost-efficient strategies that can be leveraged to help youth adhere to the actions in the HIV Prevention and Treatment Continua.

**International Registered Report Identifier (IRRID):**

DERR1-10.2196/10759

## Introduction

Adolescents represent over 20% of HIV cases in the United States [1]. Far fewer youth access the biomedical treatments available to prevent transmission and treat HIV infection compared with adults [1]. These treatments include early identification of HIV infection through repeat HIV testing, pre-exposure prophylaxis (PrEP) and postexposure prophylaxis (PEP) to prevent infection, and the use of antiretroviral (ARV) medications, or Treatment as Prevention, to reduce the risk of transmitting HIV among those living with HIV [2-4]. To stop HIV infection among adolescents, the Office of AIDS Research at the National Institutes of Health (NIH) [5] has identified a set of broadly implemented innovative intervention strategies that are consistent with the following principles:

1. The biomedical strategies to treat HIV are increasingly similar to those to prevent the acquisition of HIV; similar strategies can be successful with youth of different serostatus.
2. Syntheses of evidence-based HIV intervention programs developed over the last 30 years provide models and practices that can be used in today’s interventions.
3. Mobile technologies are an efficient strategy for sharing information, sending messages, engaging youth, and enhancing self-monitoring, regardless of the platform used.
4. Interventions should be the least intensive needed to obtain health-protective behaviors by any individual youth. A Stepped Care approach—which initially provides minimal intervention and only increases the intensity of the intervention if no change occurs—may be more effective and cost efficient than providing the same intervention package to all youth.
5. HIV prevention and treatment must be planned, tailored, and executed at a local level.
6. Our scientific breakthroughs are only relevant if we can recruit, retain, and keep youth living with HIV (YLH) and youth at high risk (YHR) of acquiring HIV engaged in health care and adherent lifelong to prevention and treatment services.

A set of 3 interrelated studies, known as the Comprehensive Adolescent Research and Engagement Studies (CARES), is being mounted to evaluate strategies to increase youth’s uptake, maintenance, and retention in the HIV Prevention and Treatment Continua [2-4].

Participants are acutely infected YLH, YLH with established HIV infection, and YHR. This paper summarizes the rationale behind these studies, including the recruitment, retention, assessment, and intervention strategies common across them. Individual protocol papers in this volume outline each specific trial [6-8]. The integrated data analytic protocols [9] and strategies for monitoring sexually transmitted infections (STI) across the studies [10] are concurrently available.

YLH and YHR in the United States are largely gay and bisexual males and transgender youth (GBTY), with black and Latino GBTY accounting for the majority of HIV diagnoses among adolescents [1,11]. In addition, young people with mental health and substance abuse disorders, those who experience homelessness and sexual abuse, and those with histories of incarceration are at increased risk for acquiring HIV [12-15]. These subgroups are overrepresented among black and Latino, low-income youth. YLH in the United States are concentrated in the South and along the West and East Coasts, with the majority of YLH living in urban areas [11]. We selected Los Angeles (LA) and New Orleans—two HIV epicenters diverse in geography, the demographic distribution of HIV, and cultural characteristics—for conducting the 3 studies. By designing and testing interventions in these two cities, we aim to demonstrate the efficacy of the same intervention strategies and principles tailored to substantially different settings.

The intervention strategies for YLH and YHR are increasingly similar. Rather than creating separate programs, one team can implement services for both subgroups. ARV medications have now been demonstrated effective for both: (1) achieving HIV viral suppression, reducing risk of HIV transmission, and increasing the quality and length of life for persons living with HIV and (2) reducing acquisition of HIV among seronegative persons, that is, the success of Treatment as Prevention [2]. These breakthroughs have shifted the loci of almost all prevention and treatment services from community to medical settings. At present, 78% of federal HIV funding is in medical settings compared with 53% in 1998 [16]; this shift has two major implications for HIV prevention for youth.

**Figure 1:**
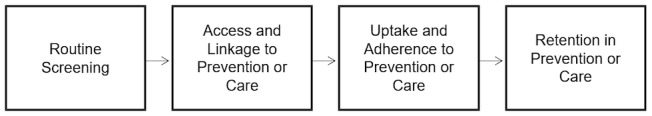
Similarity of the HIV Prevention Continua for seronegative youth at high risk for HIV and the HIV Treatment Continua for youth living with HIV.

First, the HIV Prevention Continuum and the HIV Treatment Continuum [2-4,17] are similar in the tasks that are required for youth, as shown in Figure 1. Whether a young person is a YLH or a YHR, each must be linked to medical care, have insurance to cover payments for medical care, and have the transportation and skills to navigate a medical system. In addition, optimal care for both YLH and YHR is to prescribe ARV medications, again, resulting in the need for both to adhere to medical regimens over time, have regular check-ups, and anticipate when their medications need refills. Both YLH and YHR must be monitored for issues like side effects and toxicities over time.

Because such a high percentage of YLH and YHR are GBTY, it is typical that youth must cope with stigma from others toward their sexual orientation [18]. The stigma can occur when disclosing their sexual orientation, gender identity, or HIV status to others [18]. These similarities again suggest that the interventions for these youth can be fundamentally similar.

Second, HIV services concentrated in medical settings often do not reach the youth who need them the most. Adolescents and young adults, particularly young men, do not engage in health care visits routinely, and black and Latino adolescents are less likely to utilize medical care than peers of other ethnicities [19]. YLH are more likely than adults living with HIV to be homeless, recently incarcerated, uninsured, or living in a low-income household [1]. These factors can serve as barriers to seeking medical care for HIV prevention and treatment. Street outreach programs, shelters, bars, hook-up settings, or social media sites associated with risk behaviors (eg, Grindr) are the most likely places to access these youth. Furthermore, providers are uncomfortable bringing up sexuality; 40% have difficulty bringing up the human papillomavirus vaccine, even with parents [20]. Thus, community-based recruitment efforts are likely to identify YLH and YHR in need of services, as well as medical clinic-based efforts. Therefore, we are recruiting in both community settings and adolescent medicine sites.

Although evidence-based interventions (EBIs) are difficult to replicate, they share key conceptual components [21-23]. Reviews of manuals of evidence-based adolescent HIV programs have found that all programs (1) frame the prevention message; (2) not only share knowledge regarding HIV but also help youth apply the health knowledge in their daily routines; (3) remove barriers to reaching implementing the health behavior (eg, getting insurance); and (4) build social support to sustain healthy behaviors. Based on these key conceptual components, we are evaluating 3 technology-driven intervention strategies: short message service (SMS) text messaging, peer support via social media, and paraprofessional coaching. Regardless of the delivery strategy, the intervention aims to link youth to medical care, improve access and consistent adherence to ARV medication, and increase routine health monitoring among youth. These studies test whether the strategies are also efficacious and cost effective in shaping healthy, HIV-related routines among youth.

Technology drives our approach because almost all adolescents (92%) go on the Web daily, typically with mobile devices [24]. Smartphones are increasingly available to youth, including black and Latino youth [25] and homeless youth [26]. Texting is particularly important for adolescents; 90% of those with phones text and typically receive and send 30 texts each day [24]. SMS text messages increase ARV medication adherence [27] as well as adherence to medical regimens for other chronic diseases [28]. SMS text messages change both the health-seeking and adherence behaviors of YLH and reduce risky sexual and drug use behaviors of YHR [29]. Much of this Web-based activity is driven by social media, with over 70% of adolescents aged under 18 years using Facebook, Instagram, or Snapchat [25]. Thus, technology is a powerful tool to intervene, scale, and monitor YLH and YHR to enhance HIV prevention and treatment.

A core component of our approach is an Automated Messaging and Monitoring Intervention (AMMI). Youth receive SMS text messages daily and are asked to report on their behaviors weekly. When delivering texts for ARV medication adherence, evidence shows that follow-up phone calls are needed if there is no response to a weekly text, and direct step-by-step instructions are better than vague support [30,31]. Monitoring—that is, asking youth about their lives—is directly related to a 15% change in behavior [31]. All youth in our studies receive AMMI, tailored to their personal risk factors.

Peer support via social media, detailed by Swendeman et al [8], is also used in these studies to engage and support youth. Both AMMI and social media-based peer support are important because they are scalable intervention strategies [32,33].

Paraprofessional coaching is a concept with wide cultural appeal and a more intensive interpersonal strategy to create healthy routines among YLH and YHR [34-36]. In 2 randomized controlled trials (RCTs) in sub-Saharan Africa [35,36], we found benefits lasting 5 years for mothers living with HIV and their children receiving paraprofessional coaching. Similarly, we found that paraprofessional coaching at soccer games is effective in engaging and assisting young men to reduce their HIV risk [37]. Coaches in these studies are community peers that we have trained in the key conceptual components of EBIs and in the skills that can help youth problem-solve hassles of daily living and support engagement and adherence to HIV interventions. Coaching in the CARES interventions has been detailed by Arnold et al [7].

These 3 intervention strategies utilize the same social cognitive model of behavior change. However, each uses different mediators of change. AMMI is aimed at informing, motivating, cueing or reminders, and self-monitoring. The primary change strategies for peer support are rewarding new behaviors and providing positive role models. Coaching relies on a behavior-change analyst who can link to community resources and facilitate goal-setting, problem solving, and building self-regulation skills in a real-world situation with individual youth. The intervention delivery strategies vary in resource intensity. Therefore, we are using designs that allow us to identify the minimum dosage needed by YLH and YHR to achieve their HIV-related goals. Manual-style interventions that provide the same dose, scripts, and content for all persons in the targeted population are likely to overserve the needs of many youth [23,38]. The Stepped Care model is a cost-effective and patient-centered approach to improve treatment outcomes for chronic illnesses [39]. Rather than everyone getting the same intervention, the dose and type of intervention are linked to the youth’s needs. Youth initially receive the least resource-intensive interventions and step up the intensity of the intervention only if not adhering to the medical regimen [40]. Given its clinical and financial benefits, the Stepped Care model has been used widely in the management of mental health problems, diabetes, and obesity [24,41]. We are testing its usefulness for YLH. In contrast, for the study with YHR that has a larger sample than the YLH study, we are using a randomized factorial design of the same intervention strategies to assess the efficacy and cost-effectiveness of each intervention independently and in combination.

YLH and YHR need lifelong health care, with repeated monitoring of their health status. The success of PrEP/PEP as a biomedical prevention strategy and ARV medication for treatment have demonstrated the efficacy of biomedical prevention [2,3]. Yet each of the landmark HIV studies with adults showed that only 50% adhered to the intervention [42]. Youth are typically less adherent than adults [43], especially over a long period. The HIV Prevention Continuum [17] requires regular HIV and STI testing and engagement in prevention strategies such as PrEP, PEP, and condom use. YLH must have their viral load repeatedly tested and must be monitored for concurrent STI, drug use, and other factors that impact adherence as well as for potential ARV medication-related resistance or toxicities. Ongoing counseling, support, and outreach are likely to be required over time for youth to achieve these aims. In these studies, we are recruiting and following a cohort of YHR and cohorts of YLH, linking them to medical care and other interventions, and assessing intervention efficacy at 4-month intervals over 24 months. These studies use the practices outlined by the Centers for Disease Control and Prevention (CDC) for those infected and at risk of acquiring HIV. The one deviation from CDC is that we conduct 3 HIV/STI tests annually (along with other assessments) rather than 4 times per year (due to costs).

## Methods

### Organizational Structure

Each study and the overall design were approved by review boards of the Adolescent Medicine Trials Network (ATN) and the University of California, LA (IRB#16-001372).

**Figure 2:**
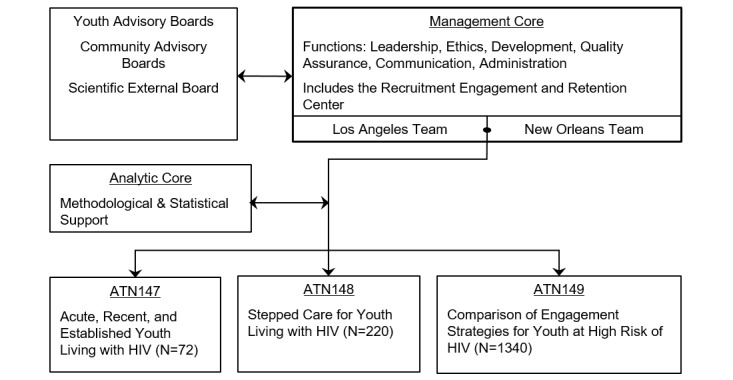
Organizational relationships between Management Core and the individual study teams, as well as the advisory board of the Comprehensive Adolescent Research and Engagement Studies program project. ATN: Adolescent Medicine Trials Network.

CARES brings together an interdisciplinary team to design, implement, and evaluate every aspect of the 3 studies. A Management Core manages the institutional review board for each study, monitors and reports adverse events; convenes Advisory Boards; manages the activities of the Recruitment, Engagement and Retention Center (RERC); and works across studies to ensure all activities are consistent with the ATN and NIH policies and procedures. The RERC has established collaborative community partnerships and supervises screening, recruitment, and retention of 1340 YHR, 220 YLH, and 72 treatment-naïve YLH who are either acutely infected or have established HIV infection. RERC staff reassess youth at 4-month intervals for 24 months. After randomization, an intervention team delivers the interventions across studies. At weekly cross-team conference calls, data on enrollment, retention, and intervention delivery are reviewed to ensure high-quality implementation. The Management Core leadership and the RERC teams interact with the Public Health and AIDS offices, and community-based and HIV care provider organizations, to provide updates on study activities on an ongoing basis. Figure 2 shows the organizational relationship of the Management Core and the 3 studies.

### Study Design

#### Adolescent Medicine Trials Network Protocol 147

This study encompasses ARV treatment initiation for acutely infected YLH and YLH with established infection. A cohort of treatment-naïve YLH will be aggressively treated with medications that are the current standard in the field and repeatedly assessed at 4-month intervals to examine if the viral reservoirs remain low, which should slow disease progression (Nielsen-Saines and Bryson, Principal Investigators). Youth will be recruited that vary in time since infection. Acute HIV infections are described as stages [44], based on the presence of viral RNA, P24 antigen, and subsequent immunoglobulin (Ig)M/IgG antibody responses. Acute infection is determined using the Fiebig stage determination based on antibody, antigen, polymerase chain reaction, and western blot results. In addition, measures of virus persistence in latent reservoirs, based on digital drop polymerase chain reaction detection of proviral DNA, estimate the replication-competent reservoir size among acutely infected YLH, which is compared with that among treatment-naïve YLH who were infected more than 90 days prior to antiretroviral therapy initiation. Additional details on Protocol 147, including power calculations for sample sizes, have been provided by Nielsen-Saines et al [6].

#### Adolescent Medicine Trials Network Protocol 148: A Stepped Care RCT for YLH With Established HIV Infection

**Figure 3:**
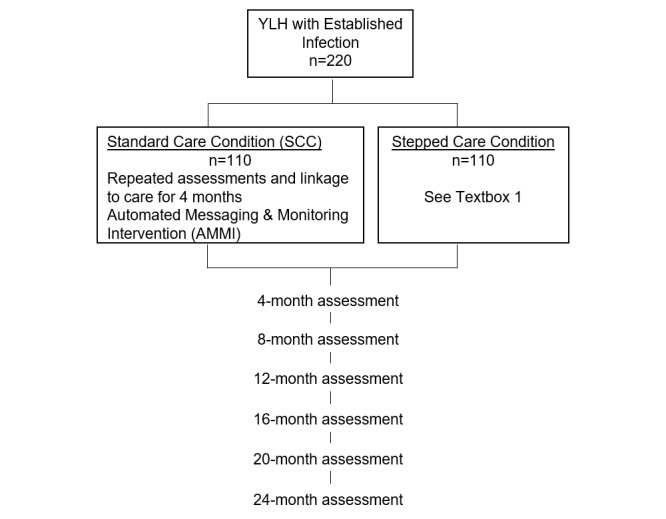
Design of the randomized controlled trial for youth living with HIV (YLH).

This study examines whether a Stepped Care approach is better than a Standard Care condition to achieve the viral suppression among treatment-experienced YLH with established infection. The 3 levels of the Stepped Care Intervention are as follows: (1) AMMI; (2) AMMI and peer support via social media; and (3) AMMI, peer support, and coaching. Figure 3 outlines the study design, and Textbox 1 lists the conditions in the Stepped Care condition. YLH in the Stepped Care arm are stepped-up to the next level of intervention if their viral load is >200 copies/mL at any 4-month follow-up assessment.

**Outline of the components in the Stepped Care Condition for youth living with HIV.**

Goals

- Linkage to care
- Adherence to antiretroviral medication
- Manage comorbidities
- Viral suppression

Level 1

- Tailored Automated Messaging Monitoring Intervention (AMMI)
- Repeat assessments for 4 months

Level 2

- Peer support
- Tailored AMMI
- Repeat assessments for 4 months

Level 3

- Coaching
- Peer support
- Tailored AMMI
- Repeat assessments for 4 months

#### Adolescent Medicine Trials Network Protocol 149: Engaging Seronegative Youth in the HIV Prevention Continuum

**Figure 4:**
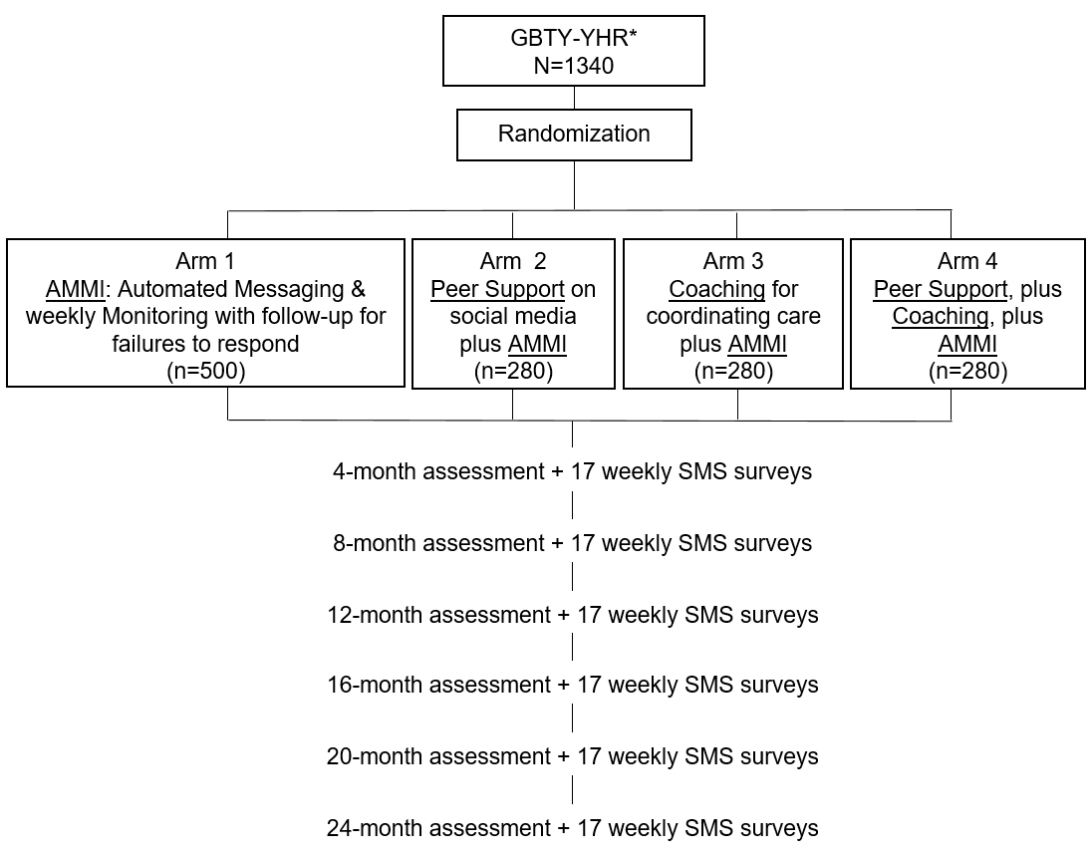
Design of the randomized controlled trial for youth at high risk (YHR) of HIV. *GBTY-YHR: Seronegative gay, bisexual, transgender youth at high risk of HIV (homeless youth); SMS: short message service.

The outcomes for this trial are as follows: staying engaged in medical care, adopting PEP after HIV exposure or PrEP prior to HIV exposure, or using condoms on 100% of sexual acts as well as repeatedly testing for HIV on an ongoing basis every 4 months. Figure 4 summarizes the following 4 active treatment conditions: (1) AMMI alone; (2) AMMI and peer support through social media; (3) AMMI and coaching; or (4) AMMI, peer support, and coaching. Because the challenges are so similar, the staff delivering the interventions—the AMMI, the peer support, and the coaches—are shared across the studies. All coaches are taught basic skills common across EBI, a theory of change based on the social cognitive model and a set of basic intervention messages. Coaches are of the same cultural backgrounds and experiences as the youth in the study, facilitating bonding and shared knowledge of difficult life circumstances. In addition, coaches and participants have access to a clinical supervisor, who is on call 24 hours, 7 days a week in each city. Furthermore, all YHR are offered transportation and an appointment for PrEP.

### Recruitment Sites

#### Sites

We selected LA and New Orleans, two HIV epicenter cities, because of their many differences. With 10.2 million persons spread across 4048 square miles, identifying and engaging YHR and YLH in LA is challenging [45]. Fortunately, LA County’s HIV epidemic and HIV-related services are concentrated in 6 communities. For example, there are 9 youth-serving agencies within 1.5 miles in the Hollywood area. However, it may take 2-3 hours to reach these agencies from different parts of LA. While New Orleans has fewer agencies than LA, the city has a better public transportation system and is much smaller, at 170 square miles, and with a population of about 393,000 persons [45]. The demographic distribution of HIV also differs between the two cities. About 70% of new HIV diagnoses in New Orleans are among black men who have sex with men (MSM) and black women [46]. About half of the new diagnoses in LA are among Latino individuals [47]. In addition, MSM account for a larger percentage of HIV diagnoses in LA (84%) [47] than in New Orleans (66%) [46].

In each city, there are collaborating agencies whose staff implement the study protocols, as well as sites at which we place our interviewers. These sites change over time based on the youth population and time of the year. The LA and New Orleans recruitment sites are listed in Textboxes 2 and 3, respectively.

In addition, recruitment is being conducted via social media in both cities. Recruiters use Grindr, Jack’d, and Scruff for targeted recruitment and Instagram, Snapchat, and Facebook to create broader visibility for the study. Recruiters post pictures and send messages via social media in real time, including while at in-person venues and recruitment events to encourage youth to approach the recruiters.

**Los Angeles recruitment sites.**

- Los Angeles LGBT Center
- Covenant House California
- The Village Family Services
- Safe Place for Youth (SPY)
- Transitional Age Youth Academy
- The Long Beach Gay and Lesbian Center
- Miller Children’s Hospital
- Multiple sites in Inland Empire of Southern California with recruitment coordinated by TruRevolution, a LGBTQ-focused community-based organization: The Riverside County Regional Medical Center, the San Bernardino Public Health Clinic, Borrego Health, Eisenhower Medical Center.

**New Orleans recruitment sites.**

- Tulane Adolescent Drop-In Center
- Tulane Adolescent Drop-In Clinic at Covenant House
- Tulane T-Cell Clinic
- University Medical Center Infectious Disease Clinic
- Crescent Care (formerly NO/AIDS Task Force)
- Brotherhood, Inc

#### Interviewers

The RERC team operates as a single entity with separate supervisors in each city. A minimum of 10 interviewers, reflective of the gender, sexual orientation, ethnicity, and life experiences of our target population, are certified as HIV test counselors and trained for 4-6 weeks on the following: phlebotomy and blood protocols; rapid diagnostic tests (RDTs; Alere, Xpert); coping with adolescents on drugs; suicide and crisis management; interview skills and role playing; HIV 101 education; study protocol; tracking and follow-up of youth; legal and ethical mandates (ie, mandated reporting of HIV and other STIs); cultural competency (GBTY and transgender-specific training); cyber bullying; housing issues; treatment of STIs for gonorrhea and chlamydia; and referral for treatment of syphilis. All contacts are documented in real time on Web-connected tablets or mobile phones using Dimagi’s CommCare platform [9]. Supervisors review data and reports from CommCare weekly and use reports in supervision meetings. There are monthly in-service trainings and random field visits to ensure high-quality work.

#### Screening

Screening for recruitment is scheduled to stagger across the days of the week and time of the day to ensure all youth receive an opportunity for participation. After obtaining verbal consent, youth are screened for study eligibility with an 18-item questionnaire conducted by the interviewer and receive a rapid point-of-care fourth-generation Alere test (Alere, Waltham, MA, USA) for HIV infection and RDT for STI as well as illicit drug use and current alcohol use. The STI testing [10] includes testing for *Chlamydia trachomatis* and *Neisseria gonorrhea* (CT/NG) using the US Food and Drug Administration-approved Xpert CT/NG assay (Cepheid, Sunnyvale, CA, USA). The Xpert CT/NG assay provides test results in 90 minutes and youth are offered same-day treatment and expedited-partner therapy in accordance with CDC recommendations. Screening for syphilis infection occurs using the Clinical Laboratory Improvement Amendments of 1988 waived rapid point-of-care fingerstick whole blood treponemal antibody test Syphilis Health Check (Diagnostics Direct, Stone Harbor, NJ, USA). Persons with reactive rapid syphilis tests are referred for having their venous serum tested for rapid plasma reagin and *Treponema pallidum* particle agglutination determination and clinical treatment.

Youth meeting the eligibility criteria are then triaged to one of the 3 studies based on the results of an initial HIV test. Protocol 147 enrolls all youth with acute or recent HIV infection and treatment-naïve YLH with established HIV infection. YLH enroll in Protocol 148 if they have had ARV medication previously. Among the 1340 YHR in Protocol 149, we anticipate that a small proportion will seroconvert and be detected during the acute infection phase and immediately triaged to enroll in Protocol 147.

### Baseline and Follow-Up Assessments

Youth in all 3 studies are assessed every 4 months over 24 months, with the RDTs used at initial screening and with a self-report interview administered face-to-face by interviewers with sensitive questions self-administered by participants using the interviewers tablet or mobile phone-based assessment app. Textbox 4 summarizes the content areas covered in the baseline and follow-up interviews. These interviews are highly similar across studies with only small variations regarding past ARV experience, adherence, and HIV-related stigma for YLH and for YHR questions on PrEP/PEP knowledge and experience.

**Domains repeatedly assessed every 4 months throughout the 24-month follow-up period for all participants in every study.**

- Rapid diagnostic tests for HIV, STI, and substance use (youth living with HIV with not be retested for HIV)
- Current health provider?
  - Current access to provider?
  - Current medical appointments?
  - Current antiretroviral (ARV) medication prescription?
  - Relationship with provider?
- Where is ARV medication prescription?
  - Drug? Dose?
  - Pharmacy?
  - Need new drug access card?
- ARV medication adherence?
- Physical health?
- Comorbidities?
  - Homelessness?
  - Mental health symptoms, care & hospitalization?
  - Drug use?
  - Alcohol use or abuse?
  - Illness? Hospitalization?
  - Gang involvement?
  - Criminal justice contact?
  - Sexual partners? Condom use?
  - Social support?
  - Employment? School?
  - Income?
  - Pleasant activities?

### Data Collection and Analysis

CommCare (Dimagi, Cambridge, MA, USA) is an open-source support program used by the research team for data collection and monitoring [9]. Automated quality assurance programs review recruitment rate, uptake of testing, HIV/STI results, intervention implementation, and responses to weekly monitoring surveys by SMS text message (or email with a weblink to a RedCap version of the survey if participants do not respond to SMS text message surveys). Reviewing these data weekly as a team across all locations, and with input from interviewers, coaches, coordinators, and principal investigators, allows for continual iterative improvement of all protocols. Senior statisticians meet monthly to review data for each protocol. Furthermore, the central analytic team supports additional researchers on projects using the deidentified data.

In addition, cost-benefit analyses are being performed on the basis of a modified form of the UNAIDS 2010 template [48]. The total costs of each intervention component of each study, as well as the costs of repeatedly assessing a cohort to facilitate engagement are monitored on an ongoing basis. Costs are of 2 types: costs of delivering the intervention and additional costs incurred by participants for their use of health care services and services from other agencies (eg, use of the criminal justice system). Intervention costs are obtained from project records and take into consideration the number of hours worked, the hourly wage, and benefits of staff. Costs to design and deliver the interventions include coaches, supervisors, facility charges, software costs, and SMS text messaging and other social media costs, messaging and mobile app data costs, additional time in coaching and supervision, and server hosting. Staff monitor their time and activity reports one week quarterly via an app to provide accurate estimates of staffing costs. The costs of additional services are derived from respondent reports of health care received, medical records, and are estimated using publicly available data. Research-specific costs (eg, incentive payments, informed consent, screens, and software adaptation for survey tools) are excluded from total costs. All cost data are price-adjusted back to year one of the study, using the medical care component of the consumer price index. These data inform not only the cost-utility analyses for this study but also future modeling studies by other researchers.

### Resource Sharing Plan

In accordance with the NIH Data Sharing Policy [49] and the NIH Public Access Policy, data, manuals, tools, and research findings generated from this study will be made publicly accessible in nonproprietary formats, free of charge, with unlimited use and distribution rights. After all data are deidentified, cleaned, and validated and main findings are published, we will make study data available to the scientific community and the general public on the Data and Specimen Hub open-source system of the National Institute of Child Health and Human Development and by the Coordinating Center of the ATN. To adhere to the “open data” quality standards, we will follow Dublin Core International metadata standards [50], following the 5-star open data deployment scheme [51].

## Results

The project is funded from September 2016 through May 2021. The study team is currently recruiting and conducting follow-up assessments. Preliminary analyses are underway. We are scheduled to complete recruitment and baseline data collection by June 2019 and expect to analyze baseline data and submit the first results for publication by fall 2019.

As of December 1, 2018, we have recruited 44 participants in Protocol 147, with 14 of these young people in the Fiebig stages 1-5. These are predominately young GBTY who are black and Latino. However, women and young heterosexual men are also included.

In Protocol 148, we have recruited 95 YLH; 68% (65/95) are black and 19% (18/95) are Latino individuals. Over half (48/95, 51%) test positive for an STI at recruitment, 67% (64/95) test positive for recent marijuana use, 10% (10/95) for cocaine, and 2% (2/95) for opiates.

In Protocol 149, 956 YHR have been recruited; 46% (440/956) are GBTY, 59% (564/956) are black, 25% (239/956) are Latino, 54% (516/956) have been homeless in the last year, 34% (325/956) have had contact with the criminal justice system, 34% (325/956) have been hospitalized for mental illness, 55% (526/956) have positive results for RDT for drug use, and 17% (163/956) have a current STI. Both YLH and YHR typically have multiple risk factors placing them at high risk for negative health outcomes.

## Discussion

This innovative set of interrelated studies provides a novel, scalable, and flexible technology-based approach for addressing the HIV epidemic among youth in the United States. These studies illustrate how currently available biomedical prevention approaches have built a “bridge” in intervention research with YLH and YHR [52], resulting in comparable intervention approaches for both groups. To achieve our goal of helping youth advance through each step of the Prevention and Treatment Continua, youth must be identified, engaged, and retained in prevention and treatment services and routinely monitored. Together the proposed studies will offer counties, cities, and communities a system for integrating and coordinating prevention and care for YLH and YHR. We expect our results will be broadly applicable to diverse agencies and jurisdictions, including the CDC, National Association of State AIDS Directors, Health Resources and Services Administration, state and local health departments, and heavily impacted communities.

One of the major innovations in this program project is to monitor the outcomes over time for youth identified and treated with ARV soon after HIV infection. Our acute infection research protocol, which has been described in detail elsewhere [6], is based on our team’s successful Cure research with pediatric populations [5]. One of the biggest challenges we face is identifying a sufficiently large pool (N=36) of youth in the acute infection phase; in the first 15 months, we have identified 13 youths. The systems that we are implementing (eg, regular HIV/STI testing, weekly automated monitoring of signs and symptoms of HIV infection), if successful, can lay the groundwork for accessing this difficult-to-identify group. Even if we identify biomedical interventions that can reduce the size of the viral reservoir and achieve a functional Cure, clinicians and researchers need a viable, cost-effective approach for identifying and engaging newly infected youth in the acute infection phase. The work we are conducting will advance our knowledge in this important area.

Another contribution of these studies is the use of technology-based approaches that emphasize the function we aim to achieve, rather than a specific platform, such as an app [53]. This approach is based on more than a decade of experience collecting data on mobile phones domestically and in resource-poor settings (South Africa, Uganda, and India), monitoring adherence daily. The technology-based interventions in these studies use off-the-shelf and rapidly deployable and adaptable tools of SMS text messaging, private social media discussion forums, and flexible communication channels for interpersonal coaching.

Cost and cost-benefit analyses are key aspects of our studies as they not only inform policy makers but also facilitate better estimates in the modeling of communities’ combination prevention strategies. Across all types of intervention studies, the costs, benefits, cost-utility, and cost-effectiveness are key issues in considering the scalability of each intervention and the value-added per dollar spent on the intervention strategy. For instance, a recent modeling experiment on the HIV Prevention Continuum for MSM in the United States found that the most successful strategy is to test MSM every 3 months for HIV infection [54]. However, the approximate cost for repeatedly testing MSM would be US $5 billion annually, a prohibitive cost and unlikely to be warranted. Unfortunately, these types of data, critical for conducting modeling exercises that can inform policy decisions, are not currently available for youth. Because we are planning to repeatedly test YHR every 4 months to approximate CDC recommendations [55] and to identify acutely infected YLH, this longitudinal dataset will yield information on the combination of risk factors among youth who go on to seroconvert. Not only will it tell us who and how to identify YLH, but it will also provide actual data for conducting modeling exercises. Finally, because no seroprevalence studies have been conducted among homeless GBTY and YHR in many years, this study informs public health administrators and policy makers whether implementing routine HIV testing in homeless shelters is warranted.

In summary, the United States is challenged to reduce new HIV infections among youth and to broadly implement the preventive interventions (particularly PEP and PrEP) that US scientists have identified. To stop HIV among youth, aggressive programs targeting YHR and YLH must be broadly implemented. This challenge requires modifications of our standard scientific approaches to replication with the fidelity of EBI, utilization of new technologies, and practical strategies for engaging and retaining youth in medical care lifelong. This set of studies examines one approach to achieving this aim.

## Abbreviations

AMMI: Automated Messaging and Monitoring Intervention
ARV: antiretroviral
ATN: Adolescent Medicine Trials Network
CARES: Comprehensive Adolescent Research and Engagement Studies
CDC: Centers for Disease Control and Prevention
CT: Chlamydia trachomatis
EBI: evidence-based interventions
GBTY: gay and bisexual males and transgender youth
LA: Los Angeles
MSM: men who have sex with men
NG: Neisseria gonorrhea
NIH: National Institutes of Health
PEP: postexposure prophylaxis
PrEP: pre-exposure prophylaxis
RCT: randomized controlled trial
RDT: rapid diagnostic tests
RERC: Recruitment, Engagement, and Retention Center
SMS: short message service
STI: sexually transmitted infection
YHR: youth at high risk
YLH: youth living with HIV
